# *BRAF*突变晚期NSCLC的靶向和免疫治疗研究进展

**DOI:** 10.3779/j.issn.1009-3419.2021.101.29

**Published:** 2021-10-20

**Authors:** 娜 李, 艳珺 徐, 云 范

**Affiliations:** 1 325035 温州，温州医科大学 Wenzhou Medical University, Wenzhou 325035, China; 2 310022 杭州，中国科学院肿瘤与基础医学研究所 Institute of Cancer and Basic Medicine (ICBM), Chinese Academy of Sciences, Hangzhou 310022, China; 3 310022 杭州，中国科学院大学附属肿瘤医院肿瘤内科 Department of Medical Oncology, Cancer Hospital of the University of Chinese Academy of Sciences, Hangzhou 310022, China; 4 310022 杭州，浙江省肿瘤医院肿瘤内科 Department of Medical Oncology, Zhejiang Cancer Hospital, Hangzhou 310022, China

**Keywords:** 肺肿瘤, BRAF, 免疫治疗, 靶向治疗, Lung neoplasms, BRAF, Immunotherapy, Targeted therapy

## Abstract

随着精准医学的发展，靶向驱动基因的治疗显著改善了晚期非小细胞肺癌（non-small cell lung cancer, NSCLC）患者的预后和生活质量。其中鼠类肉瘤病毒癌基因同源物B1（v-raf murine sar-coma viral oncogene homolog B1, *BRAF*）基因突变的NSCLC较为罕见，传统治疗遵循无驱动基因突变NSCLC的治疗方案，远远没有满足临床需求。近年来，针对*BRAF* V600E突变NSCLC的靶向治疗疗效显著，其他*BRAF*突变亚型靶向治疗仍在探索阶段。免疫疗法在*BRAF* V600E和非V600E亚型的NSCLC中也显示出积极的抗肿瘤活性。本文就*BRAF*阳性NSCLC患者的靶向和免疫治疗研究进展作一综述。

## Abstract (en)

With the development of precision medicine, therapies of targeting driver genes have significantly prolonged survival in advanced non-small cell lung cancer (NSCLC) patients. Among them, *BRAF* gene mutation is relatively rare, and the traditional regimen follows the treatment plan of NSCLC without driver gene mutation, which is far from meeting the clinical needs. In recent years, targeted therapy for NSCLC patients with *BRAF* V600E mutations has shown good efficacy when we are still exploring the better targeted therapies for other *BRAF*-mutated subtypes. Immunotherapy also showed positive antitumor activity in V600E and non-V600E subtypes of *BRAF*-mutated NSCLC. This article reviewed the progress of immunological and targeted therapy for patients with *BRAF*-mutated NSCLC.

肺癌是全球癌症中发病率和死亡率最高的癌症之一^[1]^。其中非小细胞肺癌（non-small cell lung cancer, NSCLC）的发生率为85%-90%，主要包括腺癌（占40%-50%）和鳞癌（占20%-30%）^[2]^。大约30%的NSCLC患者存在对靶向治疗敏感的基因改变[如表皮生长因子受体（epidermal growth factor receptor, *EGFR*）突变、间变性淋巴瘤激酶（anaplastic lymphoma kinase, *ALK*）重排和c-ros肉瘤致癌因子-受体酪氨酸激酶（ROS proto-oncogene 1, receptor tyrosine kinase, *ROS1*）融合]^[3]^，在肺腺癌中甚至高达45%^[4]^。随着精准医疗和基因检测技术的发展，越来越多的基因被发现与NSCLC相关，其中鼠类肉瘤病毒癌基因同源物B1（v-raf murine sar-coma viral oncogene homolog B1, *BRAF*）基因可能是NSCLC中又一个重要的驱动基因^[5]^。美国国家综合癌症网络（National Comprehensive Cancer Network, NCCN）指南已将其作为有效靶点进行常规筛查推荐^[6]^。并且，联合应用BRAF和MEK抑制剂也已成*BRAF* V600E突变NSCLC患者的标准治疗。然而对于非*BRAF* V600E突变的肺癌患者仍然遵循无驱动基因的治疗方案，缺乏有效的靶向治疗策略。免疫检查点抑制剂（immune checkpoint inhibitor, ICI）目前是另一个治疗驱动基因野生型肺癌的重要手段^[7]^。目前已有研究提示ICI治疗*BRAF*阳性的NSCLC有部分获益。本文将针对*BRAF*阳性晚期NSCLC患者的靶向和免疫治疗研究进展作一综述。

## 1 *BRAF*基因概述

### 1.1 *BRAF*基因的生物学特征

*BRAF*基因是一种重要的原癌基因，定位于7号染色体，有18个外显子，编码BRAF蛋白，与A-RAF和C-RAF同属于RAF家族^[8]^，是RAS-RAF-MEK-ERK通路的上游调节因子，参与丝裂原活化蛋白激酶（mitogen-activated protein kinase, MAPK）级联反应，在调控细胞生长和繁殖中发挥重要作用。MAPK通路信号异常主要由*RAS*和*BRAF*突变所导致，当突变发生时，BRAF蛋白磷酸化并使下游细胞外调节激酶（extracellular regulated kinases, ERK）持续激活，最终导致肿瘤的发生^[8, 9]^。所有RAF蛋白都能磷酸化MEK，但BRAF的激酶活性最高^[10]^。

*BRAF*基因突变主要发生于CR3激酶结构域的外显子11和15，根据激活RAS的信号机制和激酶活性可以将*BRAF*突变分为三个功能类别：激酶激活单体V600突变（I类）、激酶激活二聚体非V600突变（II类）和激酶受损的非V600突变异二聚体（III类）^[11]^。常见的V600E突变是第600位氨基酸上的缬氨酸（V）取代谷氨酸（E），允许BRAF作为结构性活性单体发挥作用^[12]^；非V600突变可以增加或削弱BRAF激酶活性，以CRAF介导的方式激活ERK通路^[13]^。临床前研究^[11]^提示，与I类突变相比，II类和III类*BRAF*突变更有可能与*RAS*基因突变共存，即非V600突变更有可能发生*RAS*共突变，一项大型回顾性研究也得出了这一结论。Planchard教授^[14]^在2020年美国临床肿瘤学会年会（American Society of Clinical Oncology, ASCO）的报道提示*BRAF*突变同时具有*KRAS* G13C、*cMET* 14跳读、*PIK3CA*等共突变的患者接受靶向治疗同样显示疗效。

### 1.2 *BRAF*突变的临床特征

*BRAF*基因突变是一种广谱的基因突变，最早发现于人类尤文氏肉瘤，在恶性实体瘤中的发生率约为7%^[9]^，恶性黑色素瘤中约为50%^[8]^、甲状腺乳头状癌为36%-53%、散发性结直肠癌为5%-22%，低级别卵巢浆液性癌约为30%^[15]^。*BRAF*突变在肺癌中的检出率为1%-3%，以肺腺癌多见^[16]^，在不同国家和人种中没有显著差异^[17-19]^，其中V600E突变和非V600E突变各占*BRAF*突变肺癌的50%^[20]^。

一项回顾性研究分析了1, 837例*EGFR*突变NSCLC患者的临床和分子特征，发现在相同驱动基因的NSCLC患者中，不同的突变亚群可能具有不同的临床特征，并且他们从相同治疗中获益的程度可能存在差异^[21]^。另有一项纳入了1, 046例NSCLC患者的回顾性研究发现，*BRAF* V600E突变肺癌患者多为女性、不吸烟者，且80%的V600E突变肿瘤具有微乳头特征，与较短的无病生存期（disease-free survival, DFS）和总生存期（overall survival, OS）显著相关，而非V600E突变者均有吸烟史，与预后无明显相关性^[17, 18]^。最近的一项荟萃分析^[22]^报告了*BRAF*突变NSCLC的临床特征，纳入了16项研究共11, 711例NSCLC患者，研究提示女性患者中的*BRAF*突变率略高于男性（3.02% *vs* 2.43%, *P*=0.02），但这与另一项研究^[23]^中的荟萃分析结果相反，而V600E突变患者多无吸烟史，这与以往的研究结果相同。以上回顾性研究提示，与*BRAF*非V600E突变相比，V600E突变与NSCLC不良预后相关，一线化疗的DFS和OS较短，但两者在性别、年龄方面无显著差异^[24]^。2012年-2013年法国国家癌症中心的一项晚期NSCLC分子筛查项目（*n*=17, 664）对其中的230例*BRAF*突变患者进行了为期1年的治疗效果跟踪评估^[25]^，结果发现以化疗为主的这部分人群一线缓解率仅为23%，二线缓解率仅为9%，且与*EGFR*阳性或*ALK*阳性人群相比，其中位OS及无进展生存期（progression-free survival, PFS）均较短，这项结果显示*BRAF*突变NSCLC患者以化疗为主的治疗预后较差。

### 1.3 *BRAF*突变的检测

目前NCCN指南推荐的*BRAF*突变检测方法有Sanger测序、实时聚合酶联反应（real-time polymerase chain reaction, RT-PCR）、二代测序（next-generation senquencing, NGS）等。Sanger测序属于一代测序，主要用于点突变和小变异突变，可以检测V600突变及罕见突变，但其灵敏度较低，无法检测到染色体拷贝数和易位的变化^[26]^，对肿瘤细胞的含量要求较高（> 20%）^[27]^。RT-PCR是一种能够检测已知突变的有针对性的方法，它的检测下限较低，能检测7%的突变DNA拷贝数，且价格低廉，灵敏度达到97.5%，对于V600E和V600K的检出率较高，主要缺点是罕见的*BRAF*突变亚型经常被遗漏^[28, 29]^。NGS能够同时进行多基因检测，包括所有可能的变异类型，比如突变、插入和扩增，其DNA检测下限为5%，可以获得更广泛的分子图谱，确定患者可能获得靶向治疗的其他罕见基因驱动突变，缺点是耗时较长^[30]^。免疫组化（immunohistochemistry, IHC）是检测*BRAF* V600E突变的另一个可选方法，最常用的抗体是单克隆抗体VE1，具有较高的敏感性和特异性^[31]^，对于所有*BRAF*非V600E突变的检测特异性也能达到100%。它的主要缺点在于由于*BRAF* V600E的高度异质性或低丰度可能出现假阴性，并且目前缺乏*BRAF* V600K或其他变异体的抗体，这种诊断测试不能确定是否存在其他突变。此外，由于IHC识别蛋白质而不是DNA，因此IHC和基于DNA的检测结果之间可能存在差异^[28]^。上述检测方法均以组织活检为基础，但近年来液体活检发展迅速，成为许多无法获取组织标本患者的替代选择。但由于血液缺乏解剖学特异性，血液中*BRAF*突变DNA的存在并不能提示肿瘤的数量或肿瘤的来源，液体活检仍存在局限性^[28]^。

## 2 *BRAF*突变NSCLC的靶向治疗

BRAF阳性NSCLC患者的靶向治疗药物主要包括BRAF抑制剂和MEK抑制剂，既往的研究发现两种靶向药物联合治疗疗效优于单药^[32-34]^。

### 2.1 BRAF抑制剂

#### 2.1.1 维罗非尼（Vemurafenib）

维罗非尼是BRAF的选择性抑制剂，能有效抑制BRAF V600E的活性，半数抑制浓度（half maximal inhibitory concentration, IC_50_）可达到31 nmol/L，2011年被美国食品药物监督管理局（Food and Drug Administration, FDA）批准治疗含有*BRAF* V600E突变的不可切除或转移性恶性黑色素瘤。维罗非尼治疗NSCLC有抗肿瘤活性的第一个确凿证据来自一项针对*BRAF* V600E阳性非黑色素瘤癌症的篮子研究^[35]^。这项研究中的NSCLC队列入组了19例*BRAF* V600E阳性的经治患者，接受维罗非尼单药治疗，第8周客观缓解率（objective response rate, ORR）达到42%，中位PFS为7.3个月，12个月OS为66%。研究结果达到了预设终点，随后进行了队列扩展，共纳入62例NSCLC患者，其中包括54例经治患者和8例初治患者^[36]^。经过平均10.7个月的随访，经治患者ORR为37.0%（95%CI: 24.3%-51.3%），中位PFS为6.1个月，中位OS达到15.4个月。初治患者的ORR为37.5%（95%CI: 8.5%-75.5%），中位PFS为12.9个月，中位OS未成熟，最常见的不良反应是恶心（40%）。与经治患者相比，维罗非尼作为初治患者一线治疗时的PFS延长了1倍。

在一项回顾性EURAF队列研究中，共纳入35例*BRAF*突变晚期NSCLC患者，其中包括29例V600E突变、6例非V600E突变，接受不同的BRAF抑制剂治疗，包括维罗非尼、达拉非尼或索拉非尼^[16]^。31例患者接受一种BRAF抑制剂，4例患者前后接受了两种抑制剂。接受BRAF单药靶向治疗患者的ORR为53%（95%CI: 35.1%-70.2%），疾病控制率（disease control rate, DCR）为85%（95%CI: 68.9%-95.0%）。其中24例接受维罗非尼治疗的可评价患者，ORR为54%（95%CI: 32.8%-74.4%），DCR为96%（95%CI: 78.9%-99.9%）。总体人群的中位PFS为5个月，OS为10.8个月；V600E突变组的PFS为9.3个月，非V600E突变组PFS为1.5个月。

法国国家癌症研究所有一项关于维罗非尼的篮子试验^[37]^，以评估维罗非尼对*BRAF*阳性实体瘤的疗效和安全性。在NSCLC队列中有118例患者，包括101例*BRAF* V600突变，17例*BRAF*非V600突变，*BRAF* V600突变分别为97例V600E、2例V600K、1例V600D和1例V600M，中位随访时间为23.9个月。在*BRAF*非V600队列中，没有观察到客观反应，该队列被停止。在*BRAF* V600队列中，ORR为44.9%，中位PFS为5.2个月（95%CI: 3.8-6.8），OS为10个月（95%CI: 6.8-15.7）。以上研究均提示*BRAF* V600E突变肺腺癌患者相比非V600E突变的患者更能从靶向治疗中获益。

#### 2.1.2 达拉非尼（Dabrafenib）

达拉非尼是BRAF激酶的可逆性抑制剂，通过抑制失控的BRAF蛋白抑制肿瘤生长。达拉非尼对*BRAF*突变NSCLC患者有效，最初是在一项I期试验^[38]^中发现的，该试验招募*BRAF*突变实体瘤患者。在这项研究中，唯一的*BRAF* V600E突变NSCLC患者对该治疗有反应，后来的全球研究中发现其作用类似于维罗非尼，2013年FDA批准其用于治疗*BRAF* V600E突变黑色素瘤。

Planchard教授^[32]^发起的BRF113828 II期临床研究中，84例*BRAF* V600突变NSCLC患者接受单药达拉非尼治疗，其中包括78例经治患者和6例初治患者。结果显示达拉非尼的ORR为33%，DCR为58%，中位PFS为5.5个月，中位OS为12.7个月。84例患者中有35例（42%）发生了严重不良事件，但大多数不良反应是可耐受的。这项二期临床研究提示达拉非尼单药治疗在*BRAF* V600E阳性NSCLC患者中有一定的抗肿瘤活性，但治疗效果有限。

### 2.2 达拉非尼联合Trametinib

BRAF抑制剂单药的耐药机制可能与MAPK通路的重新激活有关，如果在BRAF抑制剂中加入MEK抑制剂，可能通过阻断ERK信号通路，从而延长了患者的疾病控制时间^[9]^。达拉非尼联合Trametinib在治疗*BRAF* V600E突变的NSCLC患者中表现出协同效应^[39]^。

BRF113928是一项达拉非尼单药或联合曲美替尼治疗*BRAF* V600E突变经治或初治晚期NSCLC患者的II期临床研究，研究分为三个队列：队列A是达拉菲尼单药组，队列B是达拉非尼与曲美替尼联合治疗经治患者组，队列C是联合治疗初治患者组。

队列B入组了57例患者，ORR达到了63.2%（95%CI: 49.3%-75.6%），中位PFS为9.7个月（95%CI: 6.9-19.6），6个月OS为65%（95%CI: 51%-76%），数据截止时，中位OS不成熟^[33]^。初治组中共36例患者接受一线达拉非尼加曲美替尼治疗，经过中位15.9个月随访，ORR为64%（95%CI: 46%-79%），DCR为75%（95%CI: 58%-88%），中位PFS为10.9个月（95%CI: 7.0-16.6），中位OS为24.6个月（95%CI: 12.3-未达），最常见的不良反应是发热^[34]^。队列C中共36例初治患者接受一线联合治疗，中位PFS为10.9个月（95%CI: 7.0-16.6），中位OS为24.6个月，ORR达到64%^[34]^。与单药BRAF靶向治疗相比，双靶联合在一线和后线治疗*BRAF* V600E突变的NSCLC中均显示出获益。基于这项研究，2017年FDA批准了BRAF-MEK双靶治疗*BRAF* V600E突变的晚期NSCLC患者^[4]^。

法国的GFPC真实世界研究^[40]^纳入了40例既往经治或未治的晚期*BRAF* V600E突变NSCLC患者，接受达拉非尼联合曲美替尼治疗。结果显示，中位PFS和OS分别为17.5个月（95%CI: 7.1-23.0）和25.5个月（95%CI: 16.6-未达）。9例接受一线治疗患者的中位PFS为16.8个月（95%CI: 6.1-23.2），中位OS为21.8个月（95%CI: 1.0-未达），31例接受二线及以上治疗的患者中位PFS和OS分别为16.8个月（95%CI: 6.1-23.2）和25.5个月（95%CI: 16.6-未达），其中不良事件导致7例患者（18%）永久停止治疗，8例患者（20%）中断治疗，研究提示达拉非尼联合曲美替尼治疗经治或未治的BRAF V600E晚期NSCLC表现出客观的有效性和可控的安全性。

在一项中国NSCLC患者的回顾性研究^[41]^中，共纳入65例患者，其中54例存在*BRAF* V600E突变，11例是非V600E突变。在接受BRAF抑制剂治疗的30例V600E突变患者中，维罗非尼单药、达拉非尼单药以及达拉非尼联合曲美替尼治疗的中位PFS分别为7.8个月、5.8个月和6个月（*P*=0.970）。对于一线化疗，V600E和非V600E患者的中位PFS相似（5.4个月*vs* 5.4个月，*P*=0.825）。这项研究提示BRAF靶向治疗对携带*BRAF* V600E突变的中国NSCLC患者有临床获益。

### 2.3 其他新药

Lifirafenib（BGB-232）是一种新型的RAF家族关键激酶和EGFR抑制剂，它对*BRAF* V600突变的实体瘤的疗效评估正处于一期临床试验阶段^[42]^。在这项一期研究中，53例*BRAF*突变实体瘤患者中，8例（15.1%）获得部分缓解（partial response, PR），27例（50.9%）为疾病稳定（stable disease, SD），结果提示Lifirafenib治疗*BRAF* V600突变实体瘤患者有一定程度的获益，但有必要进一步研究Lifirafenib单药治疗或联合使用的安全性和有效性。目前Lifirafenib联合MEK抑制剂治疗*BRAF*突变实体瘤的I期/II期试验已经开始招募（NCT03905148）。

所有的*BRAF*突变都会激活ERK磷酸化，因此推测ERK信号通路调控的转录因子是*BRAF*突变的潜在下游靶点^[9]^，BRAF和MEK抑制剂的获得性耐药可能与MAPK信号通路中ERK的重新激活有关。临床前证据也发现小分子ERK抑制剂能延缓耐药性的出现。在一项多中心I期剂量递增和扩展的临床试验（NCT01781429）^[43]^中，*BRAF*基因突变患者共91例，ERK激酶抑制剂Ulixertinib在*BRAF* V600和非V600突变的实体瘤中均显示出抗肿瘤活性，其中28例*BRAF*非V600突变中50%有客观反应。这项研究首次提供了临床证据，提示*BRAF*非V600突变可能通过下游ERK抑制而起作用，这为治疗*BRAF*非V600突变的实体瘤患者提供了新的研究靶点。

磷脂肌醇3-激酶/蛋白激酶B/雷帕霉素靶蛋白（phosphoinositide-3 kinase/protein kinase B/mammalian target of rapamycin, PI3K/AKT/mTOR）通路参与了多种实体肿瘤的发生，包括黑色素瘤、NSCLC、乳腺癌、结肠癌等，可能是BRAF-MEK靶向治疗获得性耐药机制之一。PX-866是一种不可逆异构体PI3K抑制剂，与BRAF抑制剂联合应用对黑色素瘤细胞株的增殖有协同抑制作用。Yam等^[44]^在晚期*BRAF* V600突变肿瘤患者中进行了PX-866和维罗非尼联合用药的多中心I期研究，以确定PX-866和维罗非尼的联合用药的安全性。由于这项研究没有招募到*BRAF*突变的NSCLC患者，联合用药对这类患者的疗效是未知的，但联合使用PI3K抑制剂和BRAF抑制剂可能是治疗*BRAF* V600突变肺癌患者的另一种新的尝试。

**1:** *BRAF*突变晚期NSCLC靶向治疗临床研究汇总 Summary of clinical study of targeted treatment for *BRAF*-mutant advanced NSCLC

Study	Phase	Drugs	Line of treatment	Single/Combined	Patients (*n*)	ORR (%)	PFS (mon)	mOS/OS (mon)
EURAF^[16]^	Retrospective	V	≥1	Single	35	53	5	OS: 10.8
VE-BASKET^[36]^	II	V	≥1	Single	62	37.1	6.5	mOS: 15.4
“basket”^[35]^	II	V	≥2	Single	19	42	7.3	NR
AcSé^[37]^	II	V	≥1	Single	101	44.9	5.2	OS: 10
BRF113828^[32-34]^	II	D	≥1	Single	84	33	5.5	mOS: 12.7
BRF113828^[32-34]^	II	D+T	≥2	Combined	57	63.2	9.7	NR
BRF113828^[32-34]^	II	D+T	1	Combined	36	64	10.9	mOS: 24.6
GFPC01-2019^[40]^	Retrospective	D+T	≥1	Combined	40	NR	17.5	OS: 25.5
D: Dabrafenib; T: Trametinib; V: Vemurafenib; mOS: median overall survival; BRAF: v-raf murine sar-coma viral oncogene homolog B1; ORR: objective response rate; PFS: progression-free survival; NSCLC: non-small cell lung cancer.								

## 3 *BRAF*突变NSCLC的免疫治疗

在驱动基因阳性的NSCLC患者中，ICI的活性相当弱，对于未经选择的NSCLC患者有效率为14%-20%^[45]^。因此，免疫治疗在驱动基因阳性肺癌患者中的疗效仍在探索阶段，免疫治疗在携带*BRAF*突变的NSCLC患者中的有效性也尚不明确。

IMMUNOTARGET是一项回顾性分析免疫治疗在驱动基因阳性晚期NSCLC中疗效的临床研究^[46]^。研究纳入551例各类驱动基因阳性肺癌患者，其中BRAF队列中入组了43例，中位治疗线数为二线，程序性细胞死亡受体配体1（programmed cell death ligand 1, PD-L1）中位表达水平是50%，ORR为24%，中位PFS为3.1个月，中位OS为13.6个月，这项研究提示免疫治疗对*BRAF*突变的NSCLC患者的疗效有限，*BRAF* V600E与其他*BRAF*突变的生存获益也没有统计学差异。在另一项GFPC研究^[47]^中也得出了相似的结论。

在另一项以色列多中心回顾性研究^[48]^中，入组了39例*BRAF*突变晚期NSCLC患者，队列包括21例*BRAF* V600E突变患者（A组）和18例*BRAF*非V600E突变患者（B组）。A组和B组中分别有42%和50%的病例PD-L1高表达（TPS≥50%）（*P*=0.05）。在39例患者中，22例患者（A组57%，B组55%）接受了免疫治疗（Nivolumab, *n*=11; Pembrolizumab, *n*=10; Atezolizumab, *n*=1）。结果表明免疫治疗的ORR为28%，在V600E和非V600E突变队列中免疫治疗疗效与PD-L1表达水平之间无显著相关性。这项研究的局限性是缺乏接受ICI治疗的*BRAF*野生型患者作为对照，在意大利的一项回顾性研究^[49]^中补充了这一不足，这项研究共1, 588例NSCLC患者接受Nivolumab二线治疗，BRAF状态未知人群OS为11.0个月，而*BRAF*野生型亚组和*BRAF*突变亚组的OS分别为11.2个月和10.3个月，ORR分别为9.1%和19.6%。因此，*BRAF*突变患者二线使用Nivolumab治疗疗效有限。

在2020年中国的一项多中心回顾性研究^[50]^中，收集了4, 178例患者免疫治疗的信息，探索免疫治疗在*BRAF*突变NSCLC患者中的疗效。在*BRAF*突变型和*BRAF*野生型亚组分析中，野生型的PD-L1表达高于突变型（*P*=0.198），而突变型的TMB高于野生型（*P*=0.009），但OS在两组之间没有差异（*P*=0.334）。进一步分析发现*BRAF*非V600E组的中位OS远高于*BRAF* V600E组（14个月*vs* 5个月），且达到了统计学差异（*P*=0.017）。

综上，免疫治疗在靶向治疗耐药的*BRAF*突变型患者的二线或后线治疗中，疗效与未经选择的患者无显著差异，*BRAF*突变型NSCLC患者从免疫治疗中有一定获益，但非常有限^[49]^。笔者收集了目前国内外针对或者包含*BRAF*突变NSCLC患者的靶向或者免疫治疗的临床研究，如表 2所示。所有资料均来源于https://www.chinadrugtrials.org.cn和https://www.clinicaltrials.gov。

**2:** *BRAF*突变晚期NSCLC患者的靶向或免疫临床研究概况 Summary of clinical study of targeted treatment and immunotherapy for *BRAF*-mutant advanced NSCLC

Number	Phase	Drugs	Target group	Line of treatment	*BRAF* mutations	*N*	Combined/Single	State	Targeted therapy/Immunotherapy
NCT03543306	II	D+T	Metastatic NSCLC	≥1	*BRAF* V600E	27	Combined	Recruiting	Targeted therapy
NCT04302025	II	V+C	Resectable phase II-III NSCLC	Neoadjuvant and Ajuvant	*BRAF*	60	Combined	Recruiting	Targeted therapy
NCT02974725	Ib	LXH254+LTT462，LXH254+T，LXH254+R	NSCLC and Melanoma	≥1	*BARF*/*KRAS*/*NARS*	331	Combined	Recruiting	Targeted therapy
NCT04452877	II	D+T	Metastatic NSCLC (Chinese)	1-3	*BRAF* V600E	20	Combined	Not yet recruiting	Targeted therapy
NCT02314481	II	V	NSCLC	≥1	*BRAF* V600	119	Single	Recruiting	Targeted therapy
NCT02276027	II	MEK162	Advanced NSCLC	≥1	*BRAF*/*KRAS*/*NRAS*	66	Single	Finished	Targeted therapy
NCT03178552	II/III	A+C+V	Unresectable or Advanced NSCLC	1	*BRAF* V600	700	Combined	Recruiting	Immune combined targeted therapy
NCT03049618	IIa	P+sEphB4-HSA	Locally advanced or Advanced NSCLC	≥2	*BRAF*	50	Combined	Recruiting	Immunotherapy
NCT04543188	Ia/b	ARRY461+B	NSCLC with or not with BM	≥1	*BRAF* V600	225	Combined	Not yet recruiting	Targeted therapy
R: Ribociclib; A: Atezolizumab; P: Pembrolizumab; B: Binimetinib; C: Cobimetinib; BM: brain metastases.									

## 4 探索与展望

目前BRAF抑制剂联合MEK抑制剂是治疗*BRAF* V600E突变的晚期NSCLC患者最有效的策略，且不受治疗线数的影响^[51]^。对于靶向治疗耐药的患者，免疫治疗对其有一定疗效，但获益有限，双靶治疗联合免疫治疗仍处于研究探索阶段。

尽管靶向治疗已经取得了显著的疗效，但大部分患者的耐药仍不可避免。目前对黑色素瘤的获得性耐药机制研究较多，对NSCLC患者的BRAF靶向耐药机制尚未完全阐明。在对BRAF抑制剂耐药的黑色素瘤患者中检测到了*PTEN*缺失，且具有*PTEN*缺失的患者接受BRAF抑制剂治疗的PFS更短，这或许提示*PTEN*缺失与BRAF抑制剂原发耐药有关^[51]^。有相关研究^[52-54]^报道*BRAF* V600E突变NSCLC患者靶向治疗出现耐药，涉及*KRAS* G12D、*KRAS* G12V或*NRAS* Q61K突变的出现，且最初的*BRAF* V600E驱动突变并未消失，因此RAS抑制剂联合BRAF抑制剂双靶治疗减少耐药的发生可能是一种新的研究方向。此外，*BRAF* V600E突变也被发现可能是第三代TKI奥希替尼的耐药机制之一，有病例报道使用奥西替尼耐药的*EGFR*突变患者接受达拉非尼、曲美替尼和奥希替尼三靶治疗获得了13.4个月的临床应答^[55]^，但其安全性及有效性仍有待后续大样本的研究。

目前针对*BRAF*非V600E突变的NSCLC的治疗策略尚未有系统的靶向治疗方案，化疗仍是其首选的治疗方案。一项基础研究^[24]^发现MEK抑制剂和EGFR抑制剂联合应用可以使*BRAF*非V600E突变型小鼠肺癌瘤体显著退缩。可能的机制是，在激酶活性升高的*BRAF*非V600E细胞中，EGFR通过野生型CRAF激活MAPK信号，使RAS强烈激活，但EGFR抑制剂可以中断此途径。因此，*EGFR*突变在激活MAPK信号中起关键作用，在激酶活性降低的*BRAF*非V600E突变细胞中也是MAPK信号的主要调节因子。EGFR抑制剂联合MEK抑制剂或许可以使*BRAF*非V600E患者受益，但需要进一步的临床研究证实。

## 5 总结

*BRAF*突变是NSCLC的不良预后因子，目前双靶联合是*BRAF* V600E突变晚期NSCLC的一线标准方案，对比单靶治疗、化疗和免疫治疗，双靶治疗显示出疗效和安全性上的双重优势。探索耐药机制、克服耐药机制以及研发新型的靶向药物是目前*BRAF*突变晚期NSCLC的热门研究方向。
